# Seasonal Pattern of *Batrachochytrium dendrobatidis* Infection and Mortality in *Lithobates areolatus*: Affirmation of Vredenburg's “10,000 Zoospore Rule”

**DOI:** 10.1371/journal.pone.0016708

**Published:** 2011-03-10

**Authors:** Vanessa C. Kinney, Jennifer L. Heemeyer, Allan P. Pessier, Michael J. Lannoo

**Affiliations:** 1 Department of Biology, Indiana State University, Terre Haute, Indiana, United States of America; 2 Wildlife Disease Laboratories, San Diego Zoo Institute for Conservation Research, San Diego, California, United States of America; 3 Terre Haute Center for Medical Education, Indiana University School of Medicine, Terre Haute, Indiana, United States of America; University of Minnesota, United States of America

## Abstract

To fully comprehend chytridiomycosis, the amphibian disease caused by the chytrid fungus *Batrachochytrium dendrobatidis* (Bd), it is essential to understand how Bd affects amphibians throughout their remarkable range of life histories. Crawfish Frogs (*Lithobates areolatus*) are a typical North American pond-breeding species that forms explosive spring breeding aggregations in seasonal and semipermanent wetlands. But unlike most species, when not breeding Crawfish Frogs usually live singly—in nearly total isolation from conspecifics—and obligately in burrows dug by crayfish. Crayfish burrows penetrate the water table, and therefore offer Crawfish Frogs a second, permanent aquatic habitat when not breeding. Over the course of two years we sampled for the presence of Bd in Crawfish Frog adults. Sampling was conducted seasonally, as animals moved from post-winter emergence through breeding migrations, then back into upland burrow habitats. During our study, 53% of Crawfish Frog breeding adults tested positive for Bd in at least one sample; 27% entered breeding wetlands Bd positive; 46% exited wetlands Bd positive. Five emigrating Crawfish Frogs (12%) developed chytridiomycosis and died. In contrast, all 25 adult frogs sampled while occupying upland crayfish burrows during the summer tested Bd negative. One percent of postmetamorphic juveniles sampled were Bd positive. Zoospore equivalents/swab ranged from 0.8 to 24,436; five out of eight frogs with zoospore equivalents near or >10,000 are known to have died. In summary, Bd infection rates in Crawfish Frog populations ratchet up from near zero during the summer to over 25% following overwintering; rates then nearly double again during and just after breeding—when mortality occurs—before the infection wanes during the summer. Bd-negative postmetamorphic juveniles may not be exposed again to this pathogen until they take up residence in crayfish burrows, or until their first breeding, some years later.

## Introduction

The chytrid fungus, *Batrachochytrium dendrobatidis* (Bd) [1], has been devastating amphibian populations globally [2], [3], [4], [5], [6], [7], [8], [9], but not all species [10], [11] or animals in all regions [12], [13], [14], [15] appear equally susceptible. Two scenarios for the occurrence and spread of chytridiomycosis, likely reflecting different phases of the disease course, have been proposed [16], [17]. The first is that Bd is an epidemic, spreading as a wave and wiping out individuals, populations, and species in its path. This has occurred, or is occurring in Central America, in eastern Australia, and in parts of California [18], [19], [20], [21], [22], [23], [24]. The second scenario suggests that in certain regions of the world such as North America, much of the spread of Bd occurred decades ago and that in these places it is now endemic [24], [25]. This situation may currently be the most relevant. Bd is now widespread throughout many geographic regions and is known to occur on every continent except Antarctica (where there are no amphibians); therefore, this disease may be considered global [26], [27], [28], [29], [30], [31], [32], [33], [34], [35], [36], [37], [38], [39]. A third scenario, the Bd thermal optimum hypothesis, in a sense combines the first two and has been more controversial. This hypothesis suggests widespread benign Bd distribution has been triggered to lethality by increased temperatures due to global warming [40], but there has been resistance to this idea [41].

Amphibians are the only known host for Bd [1], [24], [42]. The life history of this fungus is composed of two stages: a free-living zoospore, which is flagellated and motile in aquatic environments, and a thallus (body), which is present in amphibian skin. Thallia form zoosporangia (vesicles), which in turn produce zoospores through asexual, and perhaps sexual, reproduction [43]. Zoospores can swim about 2 cm [44] and infect keratinizing squamous epithelial cells [45]. Favorable environments, where the infection can spread, are cool and wet. Hot and dry environments are considered hostile, and temperatures >25°C may assist infected amphibians in clearing the infection [44], [46], [47]. Resistance to Bd could include one of three mechanisms, which may work singly or in combination: antimicrobial properties of skin glandular secretions [11], [48], [49], [50], [51]; antimicrobial properties of skin microflora [52], [53], [54], [55], [56], [57]; and/or immune system function [58], but this idea has been controversial [59].

Several factors complicate our attempts to understand this disease: different strains of Bd are known [60], [61], individuals in populations can gain and lose the infection seasonally [39], [62], [63], [64], and Bd-positive animals can show clinical signs of the disease (chytridiomycosis) or be completely asymptomatic [42], [60], [65], [66], [67], [68], [69], [70]. Bd infection is reported to be exacerbated by amphibian density [39], [72], tadpole longevity [39], Bd density (load dynamics) [39], Bd reservoirs [73], the presence of pesticides [74], the presence of heavy metals (in tadpoles) [75], drought [76], climate change [40], [41], and normal climatic oscillations [77]. Some amphibians, especially aquatic salamander species, African Clawed Frogs (*Xenopus laevis*), and ranids such as Bullfrogs (*Lithobates catesbeiana*), Northern Leopard Frogs (*L. pipiens*), and Rio Grande Leopard Frogs (*L. berlandieri*) are suspected to be carriers of this disease [65], [66], .

Across amphibian species, behavioral, natural history, and life history features are known to affect the course of Bd infection [78], [79], [80]. Chytridiomycosis may be most fulminant in cool, high-humidity habitats such as cloud forests and splash zones around streams [12], [81], [82]. Because Bd infects keratin-producing cells [1], [83], [84], it affects the skin of adult frogs by disrupting physiological functions such as electrolyte balance, and can be fatal [18], [45], [84], [85]. In tadpoles, however, where the skin has not yet developed keratin, Bd attacks only mouthparts [10], [86], [87], [88], [89], [90] and tadpoles can act as reservoirs for the disease [39]. During metamorphosis, Bd can spread from tadpole mouthparts to the skin and kill juveniles [18], [91]. Variations in natural history and life history features of amphibians should produce different patterns, course, and effect of Bd infection [39], [92]. To fully comprehend this disease—that is, to observe and understand variations in its life history—it will be essential to understand how Bd affects amphibians across their remarkably diverse natural history and life history patterns.

Crawfish Frogs (*L. areolatus*) are members of the *Nenirana* subgenus [93]. The other members of this group are Gopher Frogs (*L. capito*), Dusky Gopher Frogs (*L. sevosus*), and Pickerel Frogs (*L. palustris*). Both Gopher Frog species use Gopher Tortoise (*Gopherus polyphemus*) burrows, stump holes, small mammal burrows, and other retreats as refuges; Crawfish Frogs obligately utilize crayfish burrows, therefore both Gopher Frogs and Crawfish Frogs rely on other animals to create upland retreats. Given this dependence, it is no surprise that all three species are imperiled: Dusky Gopher Frogs are listed as Federally Endangered, Gopher Frogs and Crawfish Frogs have experienced sharp declines in population numbers. In Indiana, where this study was conducted, Crawfish Frogs are State Endangered.

Crawfish Frogs exhibit a notable life history/natural history pattern from the perspective of disease transmission and the broader issue of epidemiology. While Crawfish Frogs resemble most North American frogs in forming spring breeding aggregations in fishless seasonal and semipermanent wetlands, they are unique in that when not breeding they usually live singly, isolated from other Crawfish Frogs, in burrows dug by crayfish. Crawfish Frogs may occupy single burrows for long periods of time [94]. Crayfish burrows are narrow bore but deep, extending to the water table perhaps a meter or more below the soil surface [95]. During warm seasons, Crawfish Frogs occupy the upper portion of their crayfish burrow (a plow depth of 7 or 8 cm will excavate frogs) [15], either in their burrow, at the burrow entrance with their heads out, or out of their burrows on their “feeding platforms” [94]. Time-lapse photography reveals that Crawfish Frogs will spend long periods—days at a time—outside their burrows on their feeding platforms [94]. At these times frogs can be active around the clock, including the hottest portions of the hottest days of the year (>37°C). When out of their burrows, Crawfish Frogs generally do not leave the feeding platform unless to lunge at prey, and then immediately return to their feeding platform [94].

During the winter, and perhaps during the summer when rehydrating, Crawfish Frogs will sit in the water at the bottom of the burrow (JLH, unpubl.). This water is about what you would imagine it to be after a season's (or more) accumulation of frog excrement. Thompson [95] writes: “At the bottom of the frog burrows, which usually terminated at a distance of about three feet, was a mass of foul smelling clayey material containing quantities of beetle remains and considerable dead grass, the latter probably having been washed in or accidentally carried down by the frog.” When burrows are flooded following heavy rains, Crawfish Frogs will also be submerged, but in presumably cleaner water (more dilute with a reduction in solids) near the burrow entrance (JLH, unpubl.).

Here we report the first case of chytridiomycosis in Crawfish Frogs. More importantly, given the unusual natural history features of Crawfish Frogs, we describe the nature and the course of this disease in this species. We ask whether there is a life history pattern or a seasonal pattern to infection by this fungus, and whether we can determine when and where the infection is being acquired and shed. Given the tenuous conservation status of this species, we were also concerned whether chytridiomycosis is fatal to Crawfish Frogs or whether, as with other large North American ranids, they are carriers. Of course, given the idea of Vredenburg and colleagues' that an infection intensity of 10,000 zoospore equivalents leads to amphibian declines, both situations could be true [24], [39].

## Materials and Methods

### Ethics Statement

This research was conducted under IACUC number 3-24-2008 issued by Indiana State University, and Scientific Purposes License Permit number 09-0084 issued by the Indiana Department of Natural Resources. No animals were harmed while collecting Bd samples.

### Field Samples

Crawfish Frogs were handled with nitrile gloves and swabbed using cotton, wood-handled swabs. Swabs were rubbed by rolling the cotton over the body surface [42]; five rubs each on the back, sides, belly, head, between the thighs, and the bottom of each foot for a total of 50 rubs. The head of the swab was then broken off in an individually labeled 0.6 ml microcentrifuge tube (Fisherbrand 05-407-01), stored cold and shipped on ice packs prior to analysis.

#### Breeding Adults

Breeding adults were captured along drift fences [96] or in pitfall traps (buckets) adjacent to drift fences as they attempted to enter or exit two wetlands. Nate's Pond is a seasonal/semipermanent wetland with a surface area of 1,355 m^2^ and a perimeter of 208 m that usually dries by late summer; Cattail Pond is a semipermanent/permanent wetland with a surface area of 3,287 m^2^ and a perimeter of 255 m. Adults in a third wetland (Big Pond; surface area 10,146 m^2^; perimeter 573 m), too large for us to encircle with a drift fence and monitor, were captured in mesh traps.

In 2009, 66 breeding Crawfish Frogs were sampled as follows: 41 breeding Crawfish Frogs were sampled at drift fences at Nate's Pond, 21 were sampled at drift fences at Cattail Pond, and four were sampled from mesh traps deployed at Big Pond (Table 1). Breeding frogs were sampled between 31 March and 15 May. In 2010, 65 breeding Crawfish Frogs were sampled, as follows: 44 animals were sampled entering or exiting Nate's Pond, 20 frogs were sampled from Cattail, one frog was sampled from Big Pond. Sampling dates of breeding animals ranged from 12 March to 20 May. Of these animals, 14 from Nate's Pond and seven from Cattail Pond had been sampled in 2009.

**Table 1:** Number of adult Crawfish Frogs sampled per pond per year.

	Nate's	Cattail	Big	Burrow	Total breeding	Overall Total
2009	41	21	4	12	66	78
2010	44	20	1	13*	65	65
Total	85	41	5	25		

*Not including four frogs sampled as they emerged from their burrows after overwintering.

**Figure 1:**
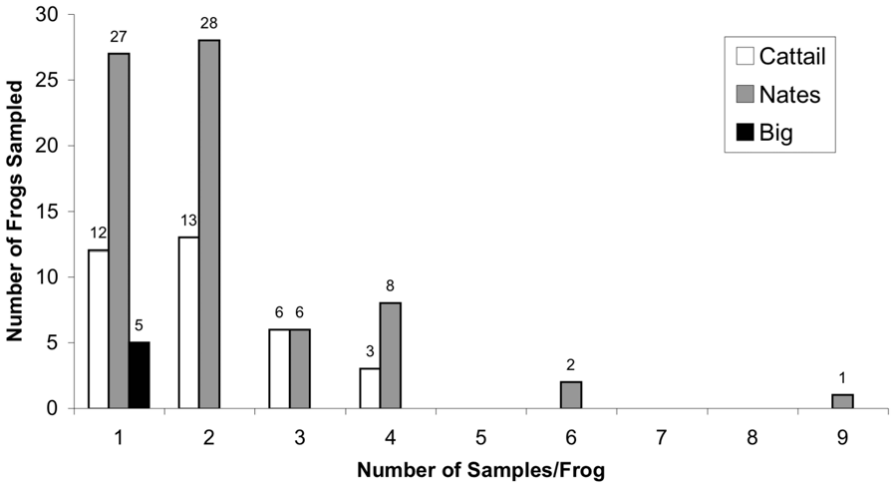
The number of times individual Crawfish Frogs were sampled for Bd across our dataset. Most frogs were sampled once or twice, one frog was sampled eight times.

After 30 March, 2009, all entering and exiting Crawfish Frog adults were sampled, except if they were handled in a way that might have contaminated the sample. An additional 12 samples were excluded due to cross-contamination during the laboratory analysis. In Nate's Pond, 154 Bd samples were analyzed from 84 frogs as follows (Fig. 1): 27 frogs were sampled once, 28 were sampled twice, six were sampled three times, eight animals were sampled four times, two animals were sampled six times, and one animal was sampled nine times. At Cattail Pond, 68 samples were analyzed from 41 frogs as follows: 12 frogs were sampled once, 13 were sampled twice, six were sampled three times, and three were sampled four times. Five animals were sampled (once) from Big Pond.

#### Upland Adults

During the summer and fall of 2009 (21 July to 21 October) and the summer of 2010 (16 April to 23 July), 25 upland Crawfish Frogs (12 in 2009, 13 in 2010; one animal each year was sampled twice) were swabbed after extricating them from their crayfish burrows [97]. Crawfish Frogs were extracted for reasons other than disease sampling, because we wished to either replace radiotransmitters, determine the status of surgical incisions following internal transmitter implantation, or determine if external belt-attached transmitters were abrading the skin.

#### Juveniles

Newly metamorphosed juveniles were captured along drift fences while exiting wetlands. In 2009, 52 juvenile Crawfish Frog samples (40/286 from Nate's, 10/11 from Cattail, two found associated with other wetlands), collected between 19 June and 17 August, were selected for analysis. In 2010, postmetamorphic juveniles were sampled randomly from 5 June to 17 July as follows. All animals sampled were from Nate's Pond; there was no Crawfish Frog metamorphosis at Cattail (VCK, unpubl.). We swabbed the first animal processed from each bucket to avoid pseudoreplication due to cross-contamination. A total of 264 swabs were taken; from these, a subsample of 99 swabs (representing 4.7% of juveniles, and 38% of swabs) were analyzed.

#### Adults Emerging from Overwintering Burrows

In 2010, four Crawfish Frog adults were sampled immediately after emerging from overwintering burrows (between 3 March and 24 March), prior to beginning their breeding migrations. These animals were either captured on the night they first emerged within mesh fences placed around burrows, or hand captured within 2 m of their burrow.

#### Post-mortem

Four adult Crawfish Frog carcasses were swabbed for Bd. One animal was found freshly killed (blood had not yet coagulated and the body was not in rigor); three had died some time (from days to weeks) prior to being sampled.

#### Other Bd samples

In 2009, samples from 15 sponges (sponges were placed, one each, in pitfall trap buckets to provide a floating substrate during bucket flooding and a source of water during dry conditions) were analyzed for the presence of chytrid. Eighteen newly-metamorphosed Marbled Salamanders (*Ambystoma opacum*), the most abundant amphibian species at our wetlands in 2009 [98], and two Smallmouth Salamanders (*A. texanum*) were also swabbed.

### Laboratory Analyses

#### PCR techniques

In 2009, Bd swabs were analyzed using conventional PCR (polymerase chain reaction) techniques [99] in the laboratory of Dr. Irene Macallister. Briefly, to extract Bd DNA from field samples, one ml of 70% ethanol was added to microcentrifuge tubes containing sample swabs and stored overnight at −20°C. Swabs were removed and the supernatant was centrifuged (16,000×g for 10 min). Then, ATL-PK (Qiagen) tissue lysis buffer (200 ml) was added to the pelleted fraction and incubated overnight (55°C). To detect Bd spores, a nested PCR approach was used [100]. Amplification products were visualized on a 3% agarose gel (Ameresco agarose 3∶1 HRB). Presence or absence of a 300-bp band was compared against the EZ Load 100-bp molecular ruler (Bio-Rad) and a positive control. Negative controls were also run with each sample; samples were analyzed twice.

Following the seasonal pattern of Bd uptake and loss detected in 2009 (see below), we decided to sample a second year (2010) using real-time Taqman PCR, a more sensitive analytical technique. In particular we were concerned about the presence of false negatives (Bd present but not detected for reasons of analytical or diagnostic sensitivity) [42]. Because we usually sampled the same individuals more than once (see below), a single negative result within a run of positive samples could either indicate acquisition, shedding, and re-acquisition of the infection, or could be the result of Bd present but not detected for analytical or diagnostic reasons. To facilitate the correct interpretation of these data, we wished to reduce the possibility of false negatives. For Taqman PCR, we followed the method of Boyle [101], [102]. Briefly, template DNA was prepared by treatment of air-dried rayon-tipped swabs (Dryswab™ Fine Tip MW113; United States: www.mwe-usa.com) with Prepman Ultra (Applied Biosystems/Life Technologies, Carlsbad, CA). PCR assays were run on a ABI/Applied Biosystems 7900HT thermocycler using 384 well plates with Applied Biosystems exogenous internal positive control labeled with Vic in separate wells to test for the presence of PCR inhibitors. For each sample, 5 uL of 1∶10 dilution (10 uL Prepman Ultra DNA extract and 90 uL water) swab DNA was added to each well with final total volume of 20 uL. Standard curves were generated with 10-fold serial dilutions (range: 10,000 to 0.001 zoospores) of laboratory cultivated *B. dendrobatidis* zoospores. With Taqman PCR, fluorescent reporter probes are used to detect Bd spores. Internal controls were run to detect the presence of PCR inhibitors. Samples were run in triplicate. Intensity of infection from Taqman PCR results was expressed as zoospore equivalents/swab.

#### Histology

Following our first suspected deaths from Bd in 2009, fresh carcasses were analyzed histologically (using conventional paraffin section and staining techniques) [103], [104], [105] for the presence of Bd [42].

## Results

### Breeding Crawfish Frogs

Over the course of 2009 and 2010, 110 individual breeding Crawfish Frogs were sampled for Bd (Table 1; several frogs were sampled across years—see below); swabs from 58 animals (53%) tested positive, as follows. In 2009, 44% (11/25) of Crawfish Frogs entering Nate's Pond were Bd positive; 37% (11/30) of animals exiting were positive (Table 2; both here and below, the numbers of animals entering and exiting wetlands are not equal due to deaths, trespassing, lost samples, ambiguous sample results, or animals simply staying in wetlands through the summer). Fifty-five percent (6/11) of animals entering Cattail Pond were Bd positive, 59% (10 out of 17) of animals exiting were positive (Table 2). In total in 2009, 47% (17/36) of Crawfish Frogs sampled upon entering wetlands tested positive for Bd; 45% (21/47) of frogs sampled upon exiting wetlands tested positive (Table 2). All four breeding adults caught in mesh traps within Big Pond were Bd positive.

**Table 2:** Rates of Bd-positive adults entering and exiting Nate's Pond and Cattail Pond in 2009 and 2010.

	2009		2010	
	Entering	Exiting	Entering	Exiting
Nate's Pond	44% (11/25)	37% (11/30)	13% (5/38)	43% (15/35)
Cattail Pond	55% (6/11)	59% (10/17)	18% (3/17)	67% (6/9)
Total	47% (17/36)	45% (21/47)	15% (8/55)	58% (21/44)

In 2010 at Nate's Pond, 13% (5/38) of Crawfish Frogs entered Bd positive; 43% (15/35) exited Bd positive. In Cattail, 18% (3/17) of Crawfish Frogs entered Bd positive; 67% (6/9) exited positive. In total, in 2010, 15% (8/55) of Crawfish Frogs entered breeding wetlands Bd positive, 58% (21/44) exited breeding wetlands Bd positive. One animal caught in a mesh trap within Big Pond was Bd negative.

Combining 2009 and 2010 data (ignoring for the moment that a subset of animals were sampled both years), 25% (16/63) of animals entering Nate's Pond were Bd positive, 40% (26/65) of animals exiting Nate's Pond were Bd positive. In Cattail Pond, 32% (9/28) of animals entering Cattail Pond were Bd positive, 62% (16/26) of animals exiting were Bd positive. Of the five breeding Crawfish Frogs captured in mesh traps at Big Pond, four (80%) were Bd positive. Overall, across both years and both drift-fenced wetlands, 27% (25/91) of Crawfish Frog adults entered breeding wetlands Bd positive; 46% (42/91) of animals exited Bd positive. From among these animals, 13 Crawfish Frogs from Nate's Pond and six from Cattail Pond were sampled at some point in both 2009 and 2010.

Of the 21 breeding Crawfish Frogs repeatedly sampled in 2009 (entering and exiting breeding wetlands), 71% (15) did not change their infection status during breeding (seven entered and exited Bd negative, eight entered and exited Bd positive); 29% (six) animals changed their status (three lost the infection, three became infected; Table 3). Among 44 breeding Crawfish Frogs sampled repeatedly in 2010, 68% (30) did not change their infection status during breeding (24 entered and exited Bd negative, six entered and exited Bd positive); 32% (14 animals) changed their status, all acquired the infection while in breeding wetlands.

**Table 3:** Summary of Crawfish Frogs arranged according to Bd infection status (Positive or Negative) as they entered and exited wetlands, by wetland and by year.

	Cattail		Nate's	
	2009	2010	2009	2010
Positive→Negative	0	0	3	0
Positive→Positive	5	2	3	4
Negative→Positive	1	4	2	10
Negative→Negative	2	5	5	19
Total	8	11	13	33

Five animals were sampled entering and exiting breeding wetlands in both 2009 and 2010 (Table 4). Of these: two animals were completely negative both years; one animal was negative except when exiting in 2009; one animal was positive entering and exiting in 2009, but negative in 2010; and one animal lost the infection while breeding in 2009 then re-acquired it during breeding in 2010.

**Table 4:** The Bd infection histories of five Crawfish Frogs, two from Cattail Pond, three from Nate's Pond, sampled in both 2009 and 2010.

	2009		2010	
	In	Out	In	Out
Cattail #1	−	−	−	−
Cattail #2	−	+	−	−
Nate's #1	+	+	−	−
Nate's #2	+	−	−	+
Nate's #3	−	−	−	−

“In” indicates entering breeding wetland, “Out” indicates exiting breeding wetland.

Over the two years of this study, 12% (5/42) of Bd infected frogs that exited wetlands developed chytridiomycosis and died. Histological examination [18], [83] of the first animal we suspected to have died from chytridiomycosis showed severe epidermal hyperplasia and hyperkeratosis with myriad chytrid thalli consistent with lethal chytridiomycosis (diagnosis confirmed by APP).

**Figure 2:**
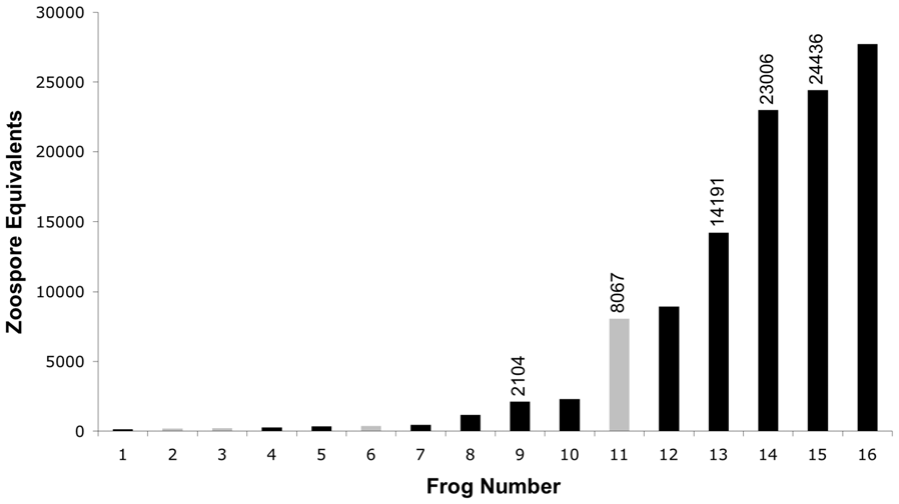
Zoospore equivalents for the 16 frogs with the highest rates of Bd infection (>100 zoospore equivalents). Values are averages of three analyses from the same swab; where multiple swabs were performed at different times on the same animal, the swab with the highest zoospore equivalents was used. Black bars are animals from Nate's Pond, gray bars are from Cattail Pond. Numbers above bars are maximum zoospore equivalents of animals that died from Bd infection. Animals with zoospore equivalents near or >10,000 that we did not find dead (numbers 12 and 16), were last observed leaving breeding wetlands. Animals with zoospore equivalents <100 did not show clinical signs of the disease.

Swabs from the five animals that died from chytridiomycosis showed consistently high infection intensity (Fig. 2), ranging from a mean of 2,104 to 24,436 zoospore equivalents. These zoospore equivalents were among the eight highest values recorded in this study (Fig. 2). We do not know the fate of the other three animals; when last seen they were exiting wetlands.

### Upland Adult Crawfish Frog Samples

All 12 adults in upland crayfish burrows sampled opportunistically during the summer of 2009, and all 13 individuals sampled during the summer of 2010, were Bd negative (Table 1). Of these animals, seven (33%) were Bd positive upon exiting breeding wetlands, and must have shed the infection or exhibited a low-level infection not detected by available PCR assays.

### Juvenile Crawfish Frog Samples

In 2009, all 52 postmetamorphic juvenile Crawfish Frogs sampled when exiting wetlands from June through August were Bd negative. In 2010, two of the 99 animals sampled tested positive. In total, 1% (2/151 juveniles) tested positive for Bd. This finding corroborates our anecdotal observations that tadpoles in our study wetlands generally have fully keratinized mouthparts (i.e., without signs of de-keratinization characteristic of Bd infection) [86], [106].

### Adults Emerging from Overwintering Burrows

Following overwintering, 50% (2/4) of Crawfish Frogs tested positive for Bd (exhibiting an infection intensity of 4 and 56 zoospore equivalents) as they emerged from their burrows.

### Post-mortem

Of the four adult Crawfish Frog carcasses sampled, one tested positive. This animal was freshly killed. The older carcasses, discovered days to weeks after death occurred, were Bd negative.

### Other Bd samples

All pitfall trap sponge samples tested Bd negative. From among the 20 ambystomatid salamanders—18 Marbled Salamanders, two Smallmouth Salamanders—sampled, 8/18 (44%) tested positive. All positive samples were from Marbled Salamanders: four from Nate's Pond, four from Cattail.

## Discussion

**Figure 3:**
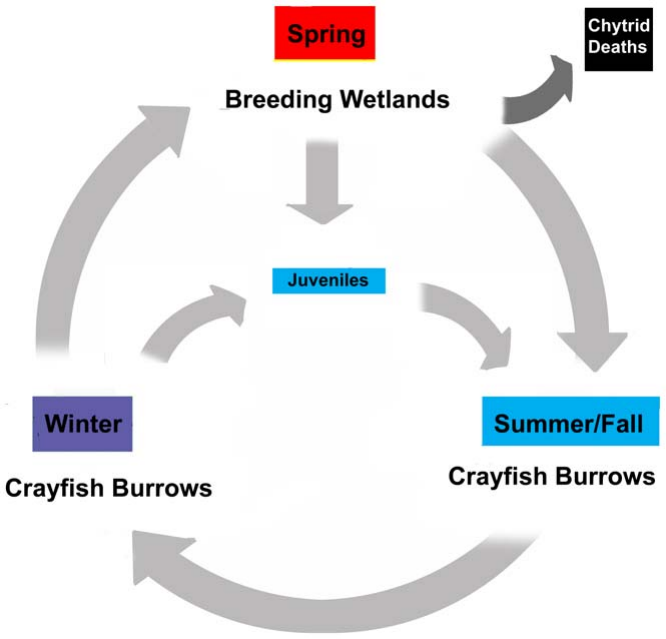
A simple model showing the patterns of Bd gain and loss in Crawfish Frogs based on our data. Red box indicates highest rate of Bd infection, blue box indicates cleared or low-level infection, purple box indicates intermediate level infection. Note that following breeding, infected frogs lose the disease during the summer when their activity is centered at the burrow entrance. During the winter, frogs inhabit the water at the base of the burrow, where a subset of animals re-acquire the disease. These infected animals then transmit Bd to their breeding wetland during relatively short (from a few hours to several days) migrations. In breeding wetlands, a subset of animals acquire Bd and a subset shed the disease, but most Crawfish Frogs maintain their status (Bd positive or negative). Some animals exiting wetlands develop chytridiomycosis and die. Exiting juvenile Crawfish Frogs were generally Bd negative (1% infection rate). Juveniles may be exposed to Bd while overwintering during the ≥two years (males) or ≥three years (females) prior to their first breeding attempts, or they may become exposed during their first breeding attempts. Once young Crawfish Frogs begin breeding, they follow the model outlined for breeding adults.

Our results and conclusions are summarized in the empirical model presented in Figure 3, and detailed here. Crawfish Frogs inhabit two distinct aquatic ecosystems that are potential sources for Bd infection: breeding wetlands, where they congregate with conspecifics as well as with other amphibian species during brief periods (days to weeks; Fig. 3, top); and crayfish burrows, where they generally live alone for most of the remainder of the year (10–11 mo; Fig. 3, bottom, right and left).

Our data suggest that Crawfish Frogs acquire Bd while overwintering in upland burrows or have low-level infections not detected by available PCR assays. While it is recommended that three tests be conducted over a 2-week period to detect all animals with low-level infections [102], because of the conservation status of Crawfish Frogs and the necessity for us to allow them to perform natural behaviors, we could not do this. Of four frogs sampled immediately upon emerging from overwintering burrows, two (50%) were Bd positive, with a low infection intensity (4 and 56 zoospore equivalents). Overall, 27% (25/91) of samples from Crawfish Frogs entering breeding wetlands on our study site were Bd positive.

Our data also suggest that Crawfish Frogs acquire Bd during breeding activities. For example, a Bd-positive female entered Nate's Pond on 8 April, 2010 with a low infection intensity (20 zoospore equivalents) and exited 15 days later with a high infection intensity (8,607 zoospore equivalents). A similar situation occurred on 19 April 2010, when a Bd-positive subadult Crawfish Frog entered Nate's Pond with 119 zoospore equivalents and exited 5 days later with 23,006 zoospore equivalents. Overall, 46% (42/91) of samples from Crawfish Frogs exiting breeding wetlands on our study site were Bd positive.

When Crawfish Frogs acquired Bd in wetlands, we do not know whether zoospores originated from the wetland substrate, directly from syntopic species of amphibians (e.g., Marbled Salamanders) [98], or whether the fungus was transmitted through zoospores spread between Crawfish Frogs during breeding-associated activities (through male-male combat or amplexus). If the latter is true, Bd-positive Crawfish Frogs entering wetlands are acting as carriers.

Twelve percent (5/42) of Crawfish Frogs sampled exiting breeding wetlands are known to have died as a result of chytridiomycosis. Survivors migrate away from wetlands and eventually into crayfish burrows. As summer proceeds, Bd-positive frogs reduce, and may lose, their infection, perhaps through behavioral thermoregulation by basking on their feeding platforms [44], [46], [47].

Sample sizes for summer, upland-dwelling Crawfish Frogs were small compared to the number of breeding adults and postmetamorphic juveniles. This could not be helped. Crawfish Frogs are among the most secretive frogs in North America [107]—it is extraordinarily unlikely that a field biologist would stumble onto and be able to sample a healthy Crawfish Frog in the open during the summer. We feel it is important that out of the 25 summer burrow-dwelling adults that were sampled (all had radiotransmitters implanted so they could be located, and were swabbed after first being extracted from burrows [97] for reasons other than disease monitoring), all were Bd negative. At a Bd infection rate of 25% (approximating the infection rate of animals entering breeding wetlands and assuming no false negatives) the probability of 21 negative samples without a positive sample is >0.001%, at an infection rate of 50% (approximating the infection rate of animals leaving breeding wetlands), the probability is much lower (5×10^−7^).

During winters, a subset of Crawfish Frogs re-acquire Bd; 27% (25/91) of frogs entering our study wetlands tested positive. While it is possible that Crawfish Frogs remain Bd free throughout the winter and instead acquire Bd during breeding migrations, we suspect they do not. Two of four animals swabbed immediately upon emerging from their overwintering burrows were Bd positive. Further, 40% (4 of 10) of telemetered Crawfish Frogs migrated from overwintering burrows to breeding wetlands using a single movement lasting one night (JLH, unpubl.). It is unlikely that a Crawfish Frog could acquire zoospores during an overnight upland migration and test positive for Bd the following morning. The remaining Crawfish Frogs used two movements to migrate from burrows to breeding wetlands; these movements were usually several days apart. When stopped during these migrations, Crawfish Frogs generally use retreats located in upland sites, often under cover of Big Bluestem (*Andropogon gerardii*) or Indian Grass (*Sorghastrum nutans*). We also feel it is unlikely that most Crawfish Frogs acquired the infection in drift fence pitfall traps (buckets). Because of the reluctance of Crawfish Frogs to move laterally along drift fences [96], we are present most nights when breeding migrations occur, and capture a large proportion of Crawfish Frogs along the fence or as they approach the fence, before they can enter buckets. Further, all samples of sponges within buckets were Bd negative.

One percent of postmetamorphic juveniles (Fig. 3, center) exit wetlands Bd positive (see [108] for data on other Midwestern species), and may not be exposed to Bd again until they take up residence in crayfish burrows, or until their first breeding (predicted to be two years later for males, three years for females) [109].

Vredenburg and colleagues [24] have suggested that a zoospore equivalent of approximately 10,000 triggers amphibian declines. Our data support this assertion. Four of our five Crawfish Frog deaths occurred in animals that exhibited zoospore equivalents near or >10,000 (frog numbers 11, 13, 14, 15; Fig. 2); the remaining frog was last sampled 15 days prior to being found dead, near the drift fence, presumably on its way back into Cattail Pond.

Cattail Pond had consistently higher rates of Bd positive animals. Cattail Pond is deeper, cooler, more permanent, and supports Green Frog (*L. clamitans*) and Bullfrog adults and larvae—potential carriers to sustain infection—throughout most years. Nate's Pond, in contrast, is shallower, warmer and semipermanent; in 2009 it dried by early September then rehydrated following heavy mid-October rains. Differences in temperature and hydroperiod may account for the differences in infection rate between animals exiting the two wetlands (a total of 40% [26/65] for Nate's, 62% [16/26] for Cattail), although these two factors would not account (at least directly) for the differences in infection rate among animals entering wetlands (25% [16/63] for Nate's, 32% [9/28] for Cattail). The overall trend both years was for Cattail Pond to have fewer breeding adult and juvenile Crawfish Frogs present, but to have a higher percentage of these animals Bd positive. In contrast, among Bd-positive animals, infection intensity, as judged by zoospore equivalents, was over four times higher at Nate's Pond (x = 4,685±8,999) than at Cattail Pond (x = 1,367±2,797), a significant difference (p = 0.01).

Following Crawfish Frog breeding and juvenile metamorphosis, Bd may be sustained in Nate's Pond (at least temporarily) and Cattail Pond (throughout most years) through the presence of the 12 other syntopic amphibian species [98]. It is more difficult to understand the persistence of Bd in the water at the base of upland crayfish burrows during the summer. Bd zoospores persist in sterilized pond water containing organic materials for as long as seven weeks, and survive at least 12 weeks in sterilized sand [110], [111]. But for Bd to be able to re-infect adult Crawfish Frogs in the bottom of their burrows when Crawfish Frogs at the entrance are Bd negative, zoospores would need to remain viable for up to six months (26 weeks). We have considered the possibility that crayfish, which can share burrows with Crawfish Frogs (JLH, unpubl.), may be transmitting the infection from wetlands to burrows, but a study demonstrating that other crustaceans (freshwater shrimp) host Bd [112] was almost immediately retracted [113]. Unlike other species of chytrid fungus, a Bd resting spore has not been identified [1]. A dormant life history stage, perhaps after sexual reproduction [43], could account for the persistence of Bd in crayfish burrows. Conversely, confinement in a small burrow might increase auto-re-infection by Bd. We can imagine a scenario where a Crawfish Frog enters hibernation with a low-level infection (perhaps undetectable by testing) or acquires the infection while overwintering. Over time, this low-level infection releases zoospores that infect adjacent skin cells on the same frog and intensity builds over time (maybe into the range detectable by testing). We plan on coupling methods to non-destructively sample water in crayfish burrows with techniques for detecting Bd in environmental samples [114], [115] to determine whether Bd is present in crayfish burrows and if so, the nature, if any, of seasonal variations in density.

Auto-re-infection may explain the differences in Bd infection rate in animals entering breeding wetlands between years. In 2010, infection rates of animals were lower at both wetlands (at Nate's Pond 44% of Crawfish Frogs were Bd positive in 2009, 13% in 2010; at Cattail Pond 55% were positive in 2009, 18% in 2010). At face value, these numbers suggest Bd was less fulminant in 2010, and this may be true. However, numbers of breeding Crawfish Frogs were substantially reduced in 2010 compared with 2009: Nate's Pond exhibited a 39% drop (69 in 2009, 42 in 2010); Cattail Pond exhibited a 25% drop (28 in 2009, 21 in 2010). It is possible that Bd prevalence was less in 2010 because Bd mortality was higher during the winter of 2009/2010— that is, animals that might usually be infected subclinically instead developed chytridiomycosis due to auto-re-infection and died. While this remains speculation, this interpretation is consistent with the observations that wetter conditions promote Bd infection, and that the fall of 2009 was unusually wet. In October, 114 mm of rain fell—28.7 mm above the 10-yr monthly average—with heavy rains coming on the 8th, 9th and 14th. These rains raised the water table to the soil surface and inundated Crawfish Frog burrows, and for much of the winter the water table remained near the soil surface [116]. We do not have enough yearly data to tie differences in Bd infection rates to environmental (temperature and moisture) conditions [117], but the data from 2009 and 2010 suggest that we might expect more annual variation in Bd infection rates in upland-dwelling frogs such as Crawfish Frogs than in aquatic frogs such as Mountain Yellow-legged Frogs [24], where water is always present and temperature extremes are moderated.

Finally, the behavior of some Bd-positive frogs at drift fences differed from the behavior of non-infected animals. Normally, Crawfish Frogs crossed our drift fences twice: once to enter wetlands prior to breeding, and once to exit after breeding. But several Crawfish Frogs repeatedly crossed our drift fences, and these animals tended to be Bd positive. In 2009, 73% (8/11) of Crawfish Frogs that crossed the fence more than twice (one entry, one exit) were Bd positive; one Bd-positive frog crossed the fence eight times (in 36 d). In 2010, 100% (6/6) of Crawfish Frogs that crossed the fence more than twice were Bd positive. We suspect the innate drive to leave wetlands following breeding was countered by the inability to osmoregulate due to chytridiomycosis [84], [85], and animals moved towards or away from wetlands depending on which urge was strongest. A subset of these animals (five) later died. One male from Big Pond was found Bd positive entering Nate's Pond on 5 May 2010; it never exited.

Crawfish Frogs in Indiana were once described as “locally plentiful” until around 1970, when many populations began to experience unexplained declines—extirpations in the absence of habitat loss [118]. We do not know what our observed annual mortality rate of 12% of breeding adults due to chytridiomyosis means to the survival of Crawfish Frog populations, but given the hypothesis of Ouellet and colleagues [25], we offer that at least a portion of these declines were due to the arrival of Bd in southwestern Indiana 40 years ago.
